# Characterization of γ‐glutamyltranspeptidases from dormant garlic and onion bulbs

**DOI:** 10.1002/fsn3.820

**Published:** 2019-01-28

**Authors:** Yuee Sun, Jing Hu, Weidong Wang, Bin Zhang, Yingbin Shen

**Affiliations:** ^1^ College of Food Science and Engineering Xuzhou Institute of Technology Xuzhou China; ^2^ Department of Food Science and Engineering Jinan University Guangzhou China; ^3^ The First Affiliated Hospital of Guangdong Pharmaceutical University Guangdong Pharmaceutical University Guangzhou China

**Keywords:** γ‐glutamyltranspeptidase, garlic, onion, purification

## Abstract

This study investigated the characteristics of γ‐glutamyltranspeptidases (GGTs) isolated from dormant garlic (*Allium sativum* L.) and onion (*Allium cepa* L. var. *agrogatum Don*) bulbs. GGTs were isolated using (NH_4_)_2_ SO_4_ precipitation and hydrophobic interaction chromatography (phenyl‐Sepharose column). The optimal temperature, optimal pH of extraction, and the effects of metal ions and organic compounds on the activity of GGTs were investigated. The optimal pH of the GGTs of garlic and onion was 5 and 7, respectively; the optimal temperatures were 70 and 50°C, respectively. Garlic's GGT had a major band at 53 kDa, whereas onion's GGT had two bands at 55 and 22 kDa. Cu^2+^, Mn^2+^, Fe^2+^, Mg^2+^, glucose, aspartic acid, and cysteine significantly enhanced the activity of garlic's GGT. Lysine and proline remarkably promoted the activity of onion's GGT, whereas Cu^2+^, glucose, and aspartic acid repress its activity. These results may deepen our understanding of allium GGTs and promote the commercial production of bioactive allium compounds.

## 1 INTRODUCTION

Phytochemicals are widely distributed in plant and are rich in teas, fruits, and vegetables (Chen, Ma, Fu, & Yan, 2017; Chen, Shen, Fu, Abbasi, & Yan, 2017). Substantial convincing evidence proves that phytochemicals possess antioxidant activity and that they play an important role in the antiproliferation of cancer cells and reduction of the risk for diabetes and cardiovascular diseases (Chen, Chen, Fu, & Liu, 2015; Chen, Zhang, Liu, Zheng, & Liu, 2016; Chen et al., 2014). Organosulfur compounds are a especially kind of phytochemicals (Liu, 2004). Generally, γ‐glutamyltranspeptidase is organosulfur compound (GGT; E.C. 2.3.2.2) and catalyzes the transfer of γ‐glutamyl groups from γ‐glutamylpeptides to other peptides, amino acids, or water. Some of the γ‐glutamylpeptides involved are bioactive organoselenium compounds and flavor precursors in allium, for example, glutamyl‐Se‐methylselenocysteine, γ‐glutamyl‐2‐carboxypropylglutathione, and γ‐glutamyl trans‐S‐1‐propenyl cysteine sulfoxide (GGPrCSO), and γ‐L‐glutamyl‐S‐alkyl‐L‐cysteine (Bayan, Koulivand, & Gorji, 2014; Dong, Lisk, Block, & Ip, 2001; Shaw, Pither‐Joyce, & McCallum, 2005), especially in garlic and onion that have anti‐disease properties (Bayan et al., 2014; Nicastro, Ross, & Milner, 2015). Furthermore, some bioactive organosulfur compounds in garlic can be formed through the removal of γ‐glutamyl group by GGT, such as S‐allyl‐L‐cysteine (Chen et al., 2015), which is a major aspect of garlic's biological activities preventing formation of free radicals, cancer, cardiovascular diseases, and neuronal degeneration diseases (Kim, 2016; Locatelli, Nazareno, Fusari, & Camargo, 2017; Pavlovic et al., 2017; Zaidi et al., 2017). In addition, GGT can participate in garlic greening and accelerate the formation of green pigment in “Laba” garlic, a traditional Chinese product (Li et al., 2008). Therefore, it is important to purify and characterize different natural GGTs for food processing or enzymatic synthesis of biological substances.

Various GGTs have been purified from *Bacillus subtilis* (Shuai, Zhang, Mu, & Jiang, 2011), tomato fruit (Martin & Slovin, 2000), Shiitake mushroom (Li et al., 2012), garlic (Zhao & Qiao, 2009), onion (Lancaster & Shaw, 1994; Shaw et al., 2005), etc. Its purification generally consists of several steps, for example, ammonium sulfate [(NH_4_)_2_SO_4_] precipitation, hydrophobic interaction chromatography, and affinity chromatography (Li et al., 2012; Shaw et al., 2005; Zhao & Qiao, 2009). Thus far, GGTs from garlic (Zhao & Qiao, 2009) and onion (Lancaster & Shaw, 1994; Shaw et al., 2005) have been partially characterized.

With a relative molecular mass of 68 kDa and a maximum absorption wavelength of 275 nm, GGT from garlic is comprised of two heterogeneous subunits (54 and 14 kDa), and its carbohydrate content is 11.31% (Zhao & Qiao, 2009). Yoshimoto et al. (2014) have identified three genes (*AsGGT1, AsGGT2*, and *AsGGT3*) that encode GGT in garlic, and the corresponding recombinant proteins can deglutamylate γ‐glutamyl‐S‐allyl‐L‐cysteine, a biosynthetic intermediate of alliin. However, there is no report on the optimum pH and temperature of GGT purified from garlic bulb or on the effects of metal ions or organic compounds on the GGT purified from garlic bulb.

Reportedly, GGT can be detected in bulbs, leaves, and roots of growing onions (Lancaster & Shaw, 1994). The GGT purified from sprouted onion bulbs has significant affinity for glutathione, and synthetic γ‐glutamyl‐p‐nitroanilide can uncompetitively inhibit the transpeptidation of GGPr CSO (Shaw et al., 2005). With a relative molecular mass of 56.7 kDa, GGT from sprouted onion bulbs shares the same N‐terminal sequence with that from Arabidopsis (Lancaster & Shaw, 1994; Shaw et al., 2005). Moreover, another GGT from onion bulb has a molecular weight of 120 kDa, optimal pH of 9.0, optimal temperature of 40°C, and activation energy of 15.8 kJ/mol (Hanum, Sinha, & Cash, 1995). A GGT from sprouted onions has an optimal pH of 9 and km of 14.3 mM for *p*‐nitroanilide per min, and it is inhibited by borate and γ‐glutamyl derivatives and activated by amino acids (Schwimmer & Austin, 1971). However, there is no investigation about the influences of metal ions or organic compounds on the activity of GGT purified from onion bulb. Moreover, no GGT has been purified from dormant onion bulbs.

To better understand the characteristics of allium GGTs, GGTs were isolated from dormant garlic and onion bulbs, and their optimal temperature and pHs were determined. In addition, the effects of metal ions and organic compounds on their catalytic properties were investigated. This study seeks to expand our understanding of GGTs from allium and provide data on the synthesis in vitro of bioactive compounds of allium.

## 2 MATERIALS AND METHODS

### 2.1 Materials

Dormant garlic (*Allium sativum* L.) and onion (*Allium cepa L. var.agrogatum Don*) bulbs were purchased from a local market in August and October of 2014, respectively. A phenyl‐Sepharose column was obtained from Pharmacia (NJ, USA). L‐γ‐glutamyl‐p‐nitroaniline, L‐methionine, p‐nitroaniline, and β‐mercaptoethanol were obtained from Sigma‐Aldrich (MO, USA), whereas other chemicals were manufactured by Sinopharm Chemical Reagent Co. Ltd (Shanghai, China; analytical grade).

### 2.2 Preparation of GGTs from garlic or onion bulbs

After removing the dry outer membranaceous scales, bulb tissue samples were obtained from garlic and onion and stored in 4°C. Garlic (100 g) and onion (150 g) samples were used for GGT isolation by preparing a homogenate using phosphoric acid buffer (pre‐cooled, 50 mM NaH_2_PO_4_/Na_2_HPO_4_, pH7, 10% glycerol; 5% NaCl, 5 mM EDTA‐Na_2_, 25 μM pyridoxal phosphate, 1 mM PMSF [benzyl sulfonyl fluoride], and 0.05% DTT [dithiothreitol]). The homogenate was spun at 10,000 *g* in a refrigerated centrifuge for 30 min, and the supernatant was separated using the methods of (NH_4_)_2_SO_4_ precipitation and Gel filtration chromatography (Sephacryl S‐200 HR (2.5 × 42 cm^2^, GE Healthcare), elution buffer, 50 mM NaH_2_PO_4_/Na2HPO4, pH7, 1.9 ml/min), as previously described (Shaw et al., 2005; Zhao & Qiao, 2009). Sodium dodecyl sulfate‐polyacrylamide gel electrophoresis (SDS‐PAGE) with 10% polyacrylamide gels and Coomassie brilliant blue (CBB) R‐250 staining was utilized to detect the GGT purity.

### 2.3 Determination of GGT activity and protein content

γ‐Glutamyltranspeptidases activity was assayed in a 170 μl reaction system, containing 100 μl of 40 mM L‐methionine, 50 μl of 0.8 mM L‐γ‐glutamyl‐p‐nitroaniline, and 20 μl of enzyme extract. The reaction mixtures were kept at 40°C (50°C for onion) for 90 min, and the light absorption value at 405 nm (UV‐2000 ultraviolet spectrophotometer) was recorded. One enzyme unit was defined as the volume of enzyme required for the production of 1 nmol p‐nitroaniline per minute under the above condition.

Protein content was detected using CBB G‐250 staining based on bovine serum albumin standard curve.

### 2.4 Analysis of optimum pH and temperature of GGTs

The optimum pH and temperature were determined by performing the GGT activity assay in different buffers (pH 3–11 for garlic GGT and pH 4–12 for onion GGT; reaction time: 90 min) at different temperatures (temperature: 20–70°C; reaction time: 30 min). In the optimization of pH, 50 mM Tris–HCl was used as the buffer for pH 9 analysis, Na_2_CO_3_–NaHCO_3_ was used as the buffer for pH 10 analysis, whereas a solution composed of sodium phosphate, HCl, and NaOH was used as the buffer for other pH analyses. In the optimization of temperature, 50 mM sodium phosphate (pH7) was used as the buffer for garlic GGT.

### 2.5 Effects of metal ions on GGT activity

To study the effects of metal ions on GGT activity, ions such as Cu^2+^ (CuSO_4_), Mn^2+^ (MnSO_4_), Zn^2+^ (ZnSO_4_), Ca^2+^ (CaCl_2_), Fe^2+^ (FeSO_4_), and Mg^2+^ (MgSO_4_) at 2 mM concentrations were added separately to the GGT activity assay system. In the control reaction, no extra metal ion was added into the reaction system, and 30 mM Tris–HCl (pH7) was utilized as the buffer for all reactions.

### 2.6 Effects of organic compounds on GGT activity

To study the effects of organic compounds on GGT activity, compounds such glucose (Glc; 1%), glycine (Gly; 10 mM), glycine methyl ester (GME; 10 mM), lysine (Lys; 10 mM), methionine (Met; 10 mM), aspartic acid (Asp; 5 mM), cysteine (Cys; 10 mM), and proline (Pro; 10 mM) were added individually to the reaction system. In control reaction, no any extra compound was added to the reaction system, and 30 mM Tris–HCl (pH 7) was used as the buffer for all reactions.

### 2.7 Statistical analysis

With 3 replicates, data were shown as means ± standard deviation, which were analyzed using SPSS software (version 19.0). Figures were drawn using Origin software (version 8.0) and assembled using Adobe Illustrator CS software (version 5).

## 3 RESULTS

### 3.1 Isolation of GGT from garlic and onion bulbs

**Figure 1:**
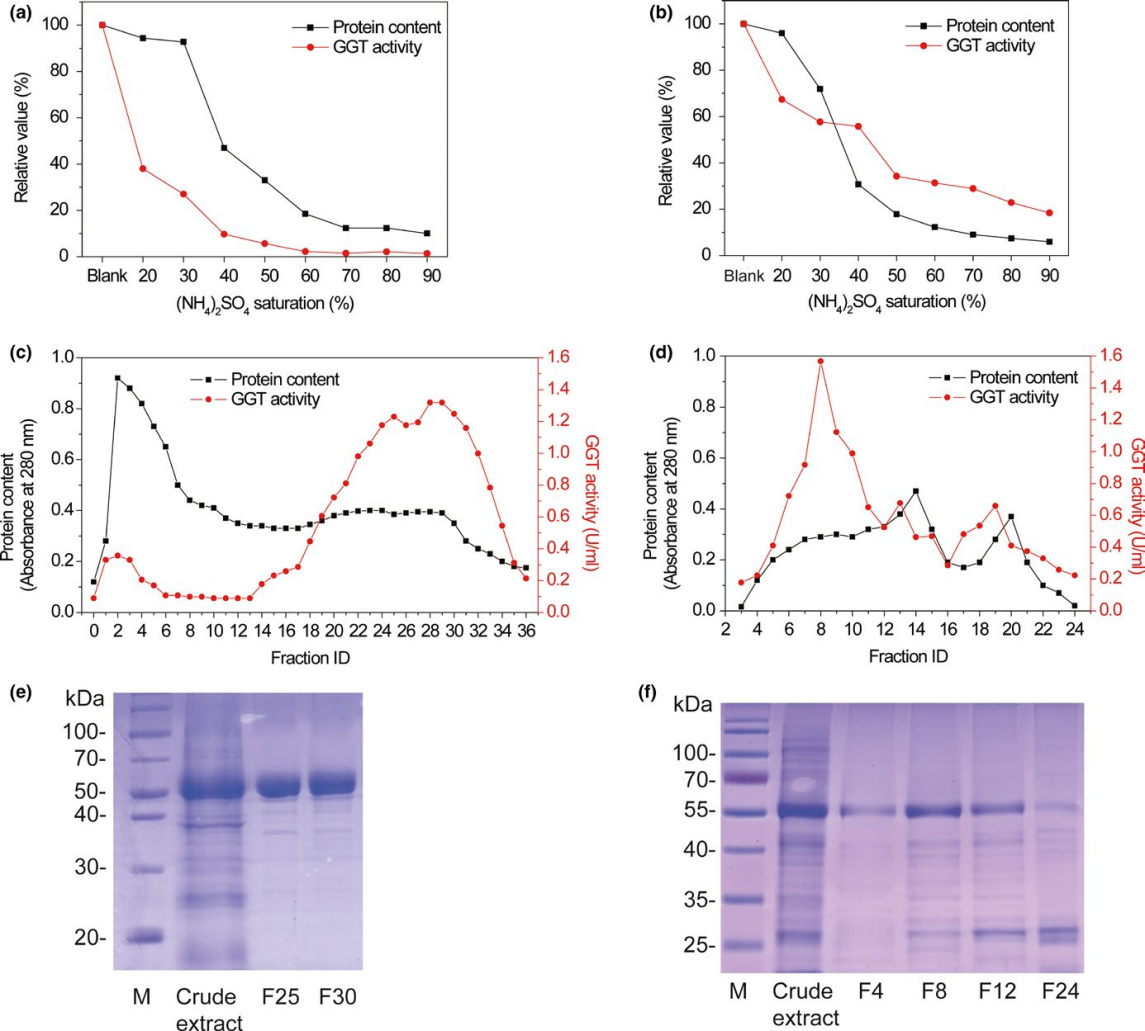
The effect of saturation (a: 60% saturation; b: 90% saturation) of (NH_4_)_2_ SO_4_ on γ‐glutamyltranspeptidases (GGTs) from garlic and onion. The fractions (c: fraction 30; d: fraction 8) of GGTs from pheny L‐Sepharose column. SDS‐PAGE analysis for the garlic's (e) and onion's (f) GGT

γ‐Glutamyltranspeptidases were successfully isolated from garlic and onion bulbs. According to the (NH_4_)_2_SO_4_ precipitation curves, 60% saturation (Figure 1a) and 90% saturation (Figure 1b) were utilized to extract crude GGTs from garlic and onion, respectively. In hydrophobic interaction chromatography, the Sepharose S‐200 HR column resolved GGT activity into fraction 30 (Figure 1c) and fraction 8 (Figure 1d) for garlic and onion, respectively. SDS‐PAGE analysis showed a major band of 53 kDa for the garlic GGT (Figure 1e), whereas SDS‐PAGE analysis showed a major band of 55 kDa and a minor band of 22 kDa for the onion GGT (Figure 1f).

### 3.2 Optimal pH and temperature of GGTs

**Figure 2:**
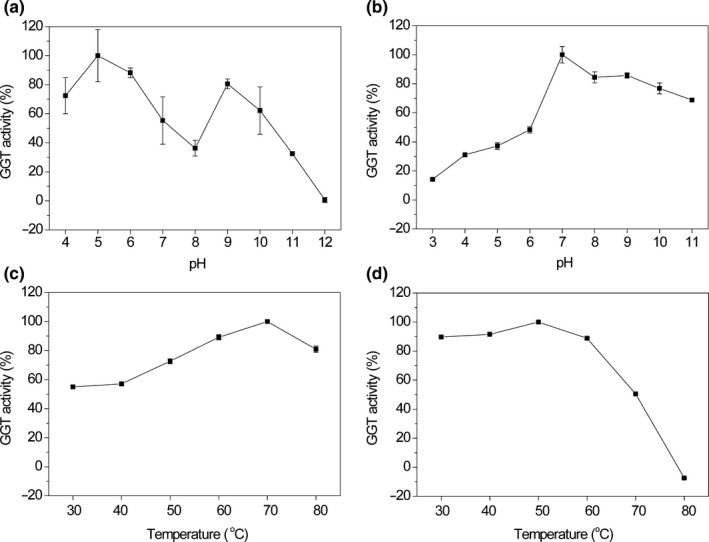
Optimal pH (a and b for garlic and onion, respectively) and temperature (c and d for garlic and onion, respectively) of GGTS

After the isolation of GGTs, optimal pH and temperature of GGTs were determined. The garlic and onion GGTs showed transpeptidase activity over a broad range of pH with optima of 5 and 7, respectively (Figure 2a and b). Especially, garlic GGT showed a “M” shape in the pH‐activity curve. In addition, the isolated garlic and onion GGTs demonstrated transpeptidase activity at a variety of temperatures with optima of 70 and 50°C, respectively (Figure 2c and d). Moreover, garlic GGT had a high activity at 60–80°C (Figure 2c), whereas onion GGT had a high activity at 30–60°C (Figure 2d).

### 3.3 Effects of metal ions on GGT activity

**Figure 3:**
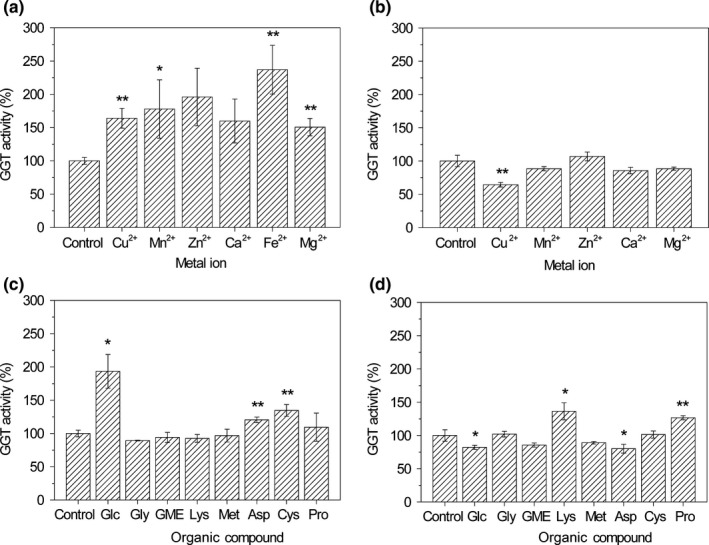
Effect of metal ions on γ‐glutamyltranspeptidases (GGT) activity for garlic (a) and onion (b); effect of organic compounds on GGT activity for garlic (c) and onion (d)

Cu^2+^, Mn^2+^, Zn^2+^, Ca^2+^, Fe^2+^, and Mg^2+^ were found to enhance the activity of garlic GGT for 64.1%, 77.9%, 95.9%, 59.8%, 137.0%, and 50.6%, respectively; however, only Cu^2+^(*p*‐value <0.01), Mn^2+^(*p*‐value <0.05), Fe^2+^ (*p*‐value <0.01), and Mg^2+^ (*p*‐value <0.01) had significant effects (Figure 3a). In contrast, Cu^2+^ (*p*‐value <0.01) was identified to significantly inhibit the activity of onion GGT for 35.6%, and Mn^2+^, Zn^2+^, Ca^2+^, and Mg^2+^ had no distinct effects (Figure 3b). Moreover, when Fe^2+^ (FeSO_4_) was added to the reaction system of onion GGT, precipitate was formed.

### 3.4 Effects of organic compounds on GGT activity

Glc, Asp, and Cys were found to significantly promote the activity of garlic GGT for 93.5% (*p*‐value <0.05), 20.5% (*p*‐value <0.01), and 34.7% (*p*‐value <0.01), respectively (Figure 3c). Glc and Asp significantly suppressed the activity of onion GGT for 17.8% (*p*‐value <0.05) and 19.9% (*p*‐value <0.05), respectively. On the contrary, Lys and Pro enhanced the activity of onion GGT for 36.3% (*p*‐value <0.05) and 26.7% (*p*‐value <0.01), respectively (Figure 3d).

## 4 DISCUSSION

γ‐Glutamyl peptide metabolism plays crucial roles in the metabolism and biosynthesis of xenobiotics and secondary products in allium (Bayan et al., 2014; Dong et al., 2001; Shaw et al., 2005). The key catalytic enzymes transferring γ‐glutamyl are GGTs. However, the properties of GGTs from allium were not fully characterized, especially GGTs from garlic and onion. In this study, the isolated GGTs from dormant garlic and onion bulbs were characterized.

SDS‐PAGE analysis showed a major band of 53 kDa for the garlic GGT, indicating that it had a molecular weight of 53 kDa. However, a major band of 55 kDa and a minor band of 22 kDa were shown for the onion GGT. Previous studies demonstrated that GGT generally consists of a major subunit and a minor subunit (Shaw et al., 2005; Zhao & Qiao, 2009). Zhao and Qiao (2009) had found that GGT from garlic bulb is composed of a 54‐kDa subunit and a 14‐kDa subunit, and Li et al. (2012) had identified that GGT purified from Shiitake mushroom (*Lentinus edodes*) is a heterodimer consisting of a 60‐kDa subunit and a 28‐kDa subunit. Moreover, GGT from sprouted onion consists of a large subunit of 36–39 KDa and a small subunit of 25 KDa (Shaw et al., 2005). These reports are different from our results, demonstrating that the GGT purified from garlic bulbs in this study is a new GGT with specific molecular component. Moreover, it is declared that GGT can only be detected in sprouted or growing onion rather than dormant onion bulbs (Lancaster & Shaw, 1994). In our study, a GGT was purified from dormant onion bulbs, and it contained a 55‐kDa subunit and a 22‐kDa subunit. In addition, the GGT from dormant onion bulbs had different molecular component comparison with that from sprouted onion bulbs (Shaw et al., 2005).

In this study, we found the optimal pH of garlic and onion GGTs was 5 and 7, respectively. This is the first report demonstrating the pH optimum of garlic GGT, and the “M” shape in the pH‐activity curve is similar to that of GGT from tomato (Martin & Slovin, 2000). Moreover, the optimal pH of GGT from dormant onion bulbs is similar to that of GGT from sprouted onion (Shaw et al., 2005) or Shiitake mushroom (Li et al., 2012). In addition, the optimal temperatures of garlic and onion GGTs were 70°C (high activity: 60–80°C) and 50°C (high activity: 30–60°C), respectively. This is the first study showing the temperature optima of garlic GGT, and the temperature optima of onion GGT is similar to that of GGT from Shiitake mushroom (Li et al., 2012), agreeing with a previous study about onion GGT (Hanum et al., 1995).

Furthermore, Cu^2+^, Mn^2+^, Fe^2+^, and Mg^2+^ were found to significantly enhance the activity of garlic GGT, whereas Cu^2+^ could inhibit the activity of onion GGT. This is the first study investigating the influences of metal ions on activity of garlic and onion GGTs. Li et al. (2012) found Cu^2+^, Mn^2+^, Fe^3+^, and Mg^2+^ could inhibit the activity of GGT from Shiitake mushroom, which was completely different from our results. Generally, Mg^2+^ is an activator of various enzymes, whereas heavy metal ions can inhibit enzyme activities by binding to thiol groups of enzymes. The conflicts between our results and previous GGT study (Li et al., 2012) might be caused by the different molecular structures of GGTs from different origins.

Additionally, Glc, Asp, and Cys were found to remarkably promote the activity of garlic GGT, and Lys and Pro enhanced the activity of onion GGT. However, Glc and Asp significantly repressed the activity of onion GGT. This is the first study researching the effects of organic compounds on garlic GGT. Reportedly, some organic compounds (e.g., L‐Met, S‐propyl‐L‐cysteine, S‐methyl‐L‐cysteine) can increase the activity of onion GGT, whereas others (e.g., γ‐L‐glutamyl‐S‐methyl‐L‐cysteine, L‐glutamate, L‐glutamine, glutathione) can impede the activity of onion GGT (Schwimmer & Austin, 1971). The positive influences of Glc, Asp, Cys, Lys, and Pro on GGT activity might be caused by their functions as glutamyl acceptors, whereas the negative effects of Glc and Asp on onion GGT might be associated with their bindings to the active site of onion GGT.

Both garlic and onion belong to allium genus, which is widely used in cooking. However, their GGTs possess different properties, including molecular weights, optimal pH, optimal temperature, and activity changes under external stimuli, which may be associated with the evolution among plants in allium genus. Reportedly, GGT in fresh garlic is remarkably activated by soaking in 10 mM CaCl_2_ solution, and it catalyzes the biosynthesis of S‐allyl‐L‐cysteine (SAC) (Xu, Miao, Chen, Zhang, & Wang, 2015), a biosynthetic intermediate of alliin and allicin. Alliin is the prime organosulfur component of garlic, and allicin possesses various activities of anticancer (Chu et al., 2013; Lee, Gupta, Huang, Jayathilaka, & Lee, 2013), anti‐infection (Feng et al., 2012), anti‐diabetes (Padiya & Banerjee, 2013), and anti‐inflammation (Li et al., 2015). Therefore, the purified and characterized GGTs from garlic and onion might be further used to commercially produce bioactive compounds.

Despite the aforementioned results, this study has limitations. For instance, protein sequences of the purified GGTs are still unknown, and we will perform N‐terminal sequencing and GGT overexpressing in our future work to study the properties of the garlic and onion GGTs.

## 5 CONCLUSION

In conclusion, the GGTs isolated from dormant garlic and onion bulbs had specific properties in the aspects of molecular mass, optimal pH, and optimal temperature. Cu^2+^, Mn^2+^, Fe^2+^, Mg^2+^, Glc, Asp, and Cys enhanced the activity of garlic GGT, whereas Lys and Pro promoted the activity of onion GGT. These results may deepen our understanding of allium GGTs and accelerate the commercial production of bioactive compounds like alliin and allicin.

## CONFLICT OF INTEREST

The authors declare no conflict of interests.

## ETHICAL STATEMENT

This article does not contain any studies with human participants or animals performed by any of the authors.
